# Water Soluble Bioactives of Nacre Mediate Antioxidant Activity and Osteoblast Differentiation

**DOI:** 10.1371/journal.pone.0084584

**Published:** 2013-12-19

**Authors:** Ratna Chaturvedi, Prajjal Kanti Singha, Satyahari Dey

**Affiliations:** 1 Department of Biotechnology, Indian Institute of Technology Kharagpur, Kharagpur, West Bengal, India; 2 Department of Pathology, University of Texas Health Science Center, San Antonio, Texas, United States of America; Universidade do Porto, Portugal

## Abstract

The water soluble matrix of nacre is a proven osteoinductive material. In spite of the differences in the biomolecular compositions of nacre obtained from multiple species of oysters, the common biochemical properties of those principles substantiate their biological activity. However, the mechanism by which nacre stimulates bone differentiation remains largely unknown. Since the positive impact of antioxidants on bone metabolism is well acknowledged, in this study we investigated the antioxidant potential of a water soluble matrix (WSM) obtained from the nacre of the marine oyster *Pinctada fucata*, which could regulate its osteoblast differentiation activity. Enhanced levels of ALP activity observed in pre-osteoblast cells upon treatment with WSM, suggested the induction of bone differentiation events. Furthermore, bone nodule formation and up-regulation of bone differentiation marker transcripts, i.e. collagen type-1 and osteocalcin by WSM confirmed its ability to induce differentiation of the pre-osteoblasts into mature osteoblasts. Remarkably, same WSM fraction upon pre-treatment lowered the H_2_O_2_ and UV-B induced oxidative damages in keratinocytes, thus indicating the antioxidant potential of WSM. This was further confirmed from the *in vitro* scavenging of ABTS and DPPH free radicals and inhibition of lipid peroxidation by WSM. Together, these results indicate that WSM poses both antioxidant potential and osteoblast differentiation property. Thus, bioactivities associated with nacre holds potential in the development of therapeutics for bone regeneration and against oxidative stress induced damages in cells.

## Introduction

Nacre is comprised of an organic matrix of biomolecules embedded in crystalline calcium carbonate layers [1]. These biomolecules play crucial role in nucleation, growth induction and inhibition of nacre formation [1,2]. Several matrix proteins have been identified in the nacre and are known to play important role in mineralization. Besides regulating the mineralization process of nacre, water-soluble matrix (WSM) contributes to its biological activities [3,4]. These WSM molecules are evidently involved in cell recruitment, differentiation and stimulation to produce mineralized tissues [5,6]. Due to beneficial biological activities, nacre finds uses in several traditional pharmaceutical preparations, by stimulating bone growth and enhancing bone density. *In vivo* studies further reveal that pieces of nacre are biologically compatible when implanted in the human and animal bodies and induce bone remodeling, specifically at the interface between the nacre and bone [4,7–12]. Nonetheless, information about the factors, responsible for its biological activities remains unknown. Proteins, namely P60, P10, and PFMG3 have been identified from *Pinctada fucata*, which induce osteoblast differentiation in murine pre-osteoblast cells [13–15] However, it remains to be seen whether these bioactive molecules are represented in WSM, and if so, then whether they act individually or in harmony with other active molecules in order to induce bone differentiation. Till date, several proteins are identified in nacre from different species that induce osteoblast differentiation, suggesting their common biochemical nature. 

Besides induction of bone growth and regeneration, traditionally nacre powder is used in treatments to reduce wrinkles, aging symptoms and sunspots of the skin [16–20]. In fact, studies have shown that WSM from shell powder stimulates synthesis of extracellular matrix as well as enhances the production of factors that are implicated in cell-to-cell adhesion and communication in cutaneous fibroblasts [16,18]. In addition, it helps the skin to recover from UV damage and apoptosis [21–23]. Together, these studies indicate that WSM could act as potential antioxidant although there is no direct evidence available till date. 

Intriguingly, previous studies have also suggested that molecules with antioxidant nature could induce osteoblast differentiation. However, this remains a matter of investigation to see whether antioxidative nature of WSM contributes to its osteoblast differentiation activity. *P. fucata* is one of the best known pearl producing marine water oysters in the world and the biomolecular composition of its nacre is different from the nacre of fresh water oysters [24–26]. Hence, in this study we investigated WSM from *P. fucata* nacre for both, osteoblast differentiation activity as well as for antioxidant potential. 

## Methods

### Ethics Statement

The *in vivo* study was approved by the ‘Committee for the Purpose of Control and Supervision of Experiments on Animals’ (CPCSEA), Ministry of Environment and Forest, Government of India and Institutional Animal Ethics Committee, Indian Institute of Technology Kharagpur, India. The experiment was carried out in strict accordance with the recommended protocol provided by the committee. 

### Extraction of WSM from nacre

WSM was extracted by dissolving 100 mg finely ground powder of nacre in 100 ml of PBS. The solution was stirred overnight at 4°C and then centrifuged at 30,000 g. The supernatant was lyophilized to obtain the WSM in powdered form. This lyophilized powder was dissolved in PBS for use in bioactivity assays. 

### Dose determination of WSM in murine preosteoblast (MC3T3-E1) and human keratinocyte (HaCaT) cells

For the determination of doses that are biologically compatible for cells, cytotoxic effects of WSM on MC3T3-E1 (obtained from American Type Culture Collection, USA) and HaCaT cells (obtained from National Centre For Cell Science, Pune, India) was evaluated. MC3T3-E1 was grown in minimal essential medium alpha (αMEM), while HaCaT was maintained in Dulbecco’s minimal essential medium (DMEM) with high glucose. Culture media were supplemented with 10% heat inactivated FBS, 100 U ml^-1^ penicillin and 100 µg ml^-1^ of streptomycin. Briefly, cells were seeded in 96 well plates at a density of 1 × 10^4^ cells per well in culture media and after reaching the 70% confluency level, cells were treated with WSM in various amounts [0.006, 0.012, 0.025, 0.05, 0.1, and 0.2% (w/v)]. After 24 h of incubation, the media in the wells were removed and replaced with fresh media and incubated for another 24 h followed by MTT assay [27]. Untreated cells served as control in all sets of experiments. Percentage of cell viability was calculated with reference to cell viability in control cells. 

### Osteoblast differentiation activity of WSM

#### Alkaline phosphatase (ALP) activity assay

Bone specific alkaline phosphatase activity was assayed using an alkaline phosphatase assay kit (Cat. No. 104-LL, Sigma). In brief, confluent MC3T3-E1 cells were treated with WSM (0.005, 0.025, and 0.05% w/v) for 24 h. Cells were then harvested by trypsinization and rinsed twice with PBS. The harvested cells were lysed with 200 μl of lysis buffer (2mM MgCl_2_ and 1% Triton X-100) in a shaker for 30 min at 37°C and were sonicated. Then, 20 μl of lysate were mixed with 100 μl of p-nitrophenyl phosphate solution and were incubated at 37°C for 30 min. The reaction was stopped by adding 50 μl of 3 M NaOH and the final absorbance was measured at 405 nm in a micro plate reader. Alkaline phosphatase activity was calculated using p-nitrophenol as a standard, according to the instructions given in the kit and was expressed as ALP units’ mg^-1^ of protein min^-1^. All results were normalized by protein quantitation [28].

#### Histochemical staining for alkaline phosphatase (ALP)

Histochemical detection of ALP was performed using an ALP staining kit (Cat. No. B6555, Sigma). After 24 h treatment of WSM (0.005, 0.025, and 0.05% w/v), the cells were fixed in 10% formalin for 10 min and washed thrice with PBS (pH 7.4), before the supernatant was replaced by Tris-buffered staining solution (pH 10.4) containing BCIP/ NBT (5- bromo-4-chloro-3-indoly phosphate and nitro blue tetrazolium). After 30 min the color development was stopped by washing with PBS. The staining was examined under a phase contrast microscope and photographed (Leica Microsystems, Germany).

#### Reverse transcriptase polymerase chain reaction (RT-PCR) for bone differentiation markers: osteocalcin (OCN) and collagen-1 A2 (COL-1A2)

RT-PCR analyses were performed to monitor the transcript levels of osteoblast differentiation markers OCN and COL-1A2. Briefly, MC3T3-E1 cells were incubated with 0.025% (w/v) of WSM for 24 h and total RNA was extracted using TRI reagent. Total RNA was reverse transcribed using Thermo Script RT at 50°C for 20 min in the presence of oligo (dT) primers and RNase inhibitor. The reaction was terminated by heating at 80°C for 10 min. Reverse transcribed RNA (5 µl) containing cDNA was added to the PCR reactions, which were carried out in a final volume of 20 μl buffer containing 200 μM dNTPs, 1.5 mM MgCl_2_, 20 pmol of each forward and reverse primers, and 1U of *Thermus aquaticus* DNA polymerase. Glyceraldehyde phosphate dehydrogenase (GAPDH) was used as a housekeeping control. Primers and PCR conditions used are listed in Table **1**, as previously reported [29]. Resulting products (20 μl) were fractionated by 1.5% agarose gel electrophoresis and were stained with ethidium bromide (10 mg/ ml) for visualization of amplicons. 

**Table 1:** Primers and RT-PCR conditions used to amplify the osteoblast differentiation markers *OCN, COL-1-A2* and house-keeping gene *GAPDH*. Fw: Forward Primers, Rv: Reverse Primers.

**Gene**	**Primers**	**PCR conditions**
***OCN***	Fw:5’-AAGCAGGAGGGCAATAAGGT-3’	35 cycles: 94°C 15” , 60°C 1’ , 72°C 1’
	Rv: 5’ AGCTGCTGTGACATCCATAC-3’	
***COL-1A2***	Fw:5’-GCAATCGGGATCAGTACGAA-3’	35 cycles: 94°C 15” , 57.3°C 1’ , 72°C 1’
	RV:5’-CTTTCACGCCTTTGAAGCCA-3’	
***GAPDH***	Fw:5’-CACCATGGAGAAGGCCGGGG-3’	35 cycles: 94°C 15” , 55°C 1’ , 72°C 1’
	Rv:5’-GACGGACACATTGGGGGTAG-3’	

#### Evaluation of mineralized bone nodules formation by histochemical analysis

The deposition of hydroxyapatite i.e., bone nodule formation was examined by histochemical staining. For this, the MC3T3-E1 cells were allowed to grow to confluency. The medium was then changed to differentiation media containing α-MEM, 7% FCS, 10 mM β-glycerol phosphate and 50 μg/ ml ascorbic acid. Initial seeding density was 5 × 10^3^ cells per ml in 24 well cell culture plates. Media were replenished every two days with WSM (0.025% w/v). On day 9, cells were washed with PBS twice and analyzed for mineralized bone nodule formation by alizarin red-S [30] and von Kossa- van Gieson staining [31]. The modified van Gieson stain (for matrix-collagen) was then used as a counter stain for von Kossa staining (for mineral-phosphate) [32]. Calcification and mineral phosphate deposits were analyzed by a phase contrast microscope and were photographed (Leica, Germany).

### Antioxidant activity of WSM

#### ABTS radical cation decolorization assay

The ABTS radical cation decolorization assay was performed as reported earlier [33]. Briefly, ABTS was dissolved in PBS (pH 7.4) to 7 mM concentration, such that ABTS^+^ radical cation was produced by reaction of ABTS stock solution with 2.45 mM potassium persulfate. The reaction mixture was allowed to stand in dark at room temperature for 12-16 h before use. After 16 h, the ABTS was diluted in PBS to an absorbance value of 0.7 (± 0.02) at 734 nm and was equilibrated at 25°C. Diluted ABTS solution of 900 μl was taken in 1 ml quartz cuvette and 100 μl of WSM (0.5 to 50 mg range) were added and further incubated for 10 min. Thereafter, the absorbance was recorded at 734 nm. PBS was used as a control (100 μl) and as reagent blank (1 ml). L-ascorbic acid was used as the positive control. Decrease in absorbance value i.e. decolorization of the solution determined the scavenging efficiency of WSM and the percentage inhibition was calculated as follows: Percentage inhibition = A-B/A × 100, where A = absorbance of control, B = absorbance of test samples

#### DPPH free radical scavenging assay

The DPPH free radical scavenging assay was performed as mentioned earlier [34]. Briefly, 0.002% (w/v) of DPPH was prepared in methanol and 1 ml of this solution was mixed with 100 μl of WSM (0.5 to 50 mg range). Solution mixtures were kept in the dark for 30 min and absorbance was measured at 517 nm. PBS was used as negative control and methanol was used as reagent blank. L-ascorbic acid was used as positive control. Percentage inhibition was calculated using the same formula as described in previous section. 

#### Lipid peroxidation inhibition in mice liver homogenates by thiobarbituric acid (TBA) method

The lipid peroxidation inhibition assay was performed as described earlier [35]. Briefly, Swiss albino mice (4-5 weeks old female) were sacrificed by cervical dislocation under anesthesia and the liver homogenates were prepared. Livers were homogenized in ice-cold buffer (150 mM KCl, 50 mM Tris-HCl, pH 7.4) and were centrifuged at 12,000 g for 30 min at 4°C. Supernatants were stored in -20°C until used for lipid peroxidation inhibition assays. Lipid peroxidation was quantified by measuring malondialdehyde (MDA). A reaction mixture was prepared by addition of liver homogenates (containing 100-150 μg/ ml protein), 100 μl of WSM (0.5 to 50 mg range), 100 μM FeSO_4_, 0.1 mM L- ascorbic acid in 1 ml potassium phosphate buffer (0.2 M, pH 7.4) and were incubated at 37°C for 60 min. The reaction was stopped by adding 28% (w/v), TCA (1 ml) and 1% (w/v) TBA (1.5 ml) in succession and the solution was then heated at 100°C in a water bath for 30 min. Reaction mixture was cooled and centrifuged at 3,000 g for 15 min. The absorbance of the supernatant was measured at 532 nm. TBA reacts with MDA to form a TBARS diadduct, a pink chromogen, detectable at 532 nm. Percentage inhibition was calculated according to the formula described earlier. Alpha-tocopherol and PBS were used as positive and negative controls, respectively.

#### H_2_O_2_ induced damage of human keratinocytes and WSM treatment

The effect of WSM on H_2_O_2_ induced oxidative damage in keratinocytes was examined. Firstly, the dose of H_2_O_2_ was determined for induction of 50% cell damage. Briefly, HaCaT cells were seeded onto 96 well plates at a density of 1 × 10^4^ cells per well in DMEM high glucose medium containing 10% fetal calf serum. After reaching a 70% confluency level, cells were treated with H_2_O_2_, freshly prepared from 30% stock solution (Sigma) for different time periods (1, 6 and 12 h) in various concentrations (0.1, 0.25, 0.5, and 1mM) [36]. Post treatment, the media were replaced with fresh media and were incubated for a further 24 h. Cell viability was analyzed by MTT assay. To determine the effect of WSM on H_2_O_2_ treated HaCaT cells, 70% confluent cultures were pretreated with WSM [0.012, 0.025, and 0.05% w/v] for 24 h in the presence of 2% serum (in DMEM high glucose medium) and cells were incubated at 37°C in 5% CO_2_ humidified atmosphere. The cells were washed with PBS and media were replaced with addition of 0.2 and 0.5 mM of H_2_O_2_ for 1h. After 1h of incubation with H_2_O_2_, the media were replaced with 10% serum and were further incubated in previous conditions for 24 h. Cell viability was determined by MTT assay and the cell morphology was observed by phase contrast microscopy (Olympus).

#### UV-B induced damage of human keratinocytes and WSM treatment

The effect of WSM on UV-B induced damage in keratinocytes was evaluated. For this the cells were grown in 6 well cell culture plates. Before UV irradiation, the media was removed and cells were washed thrice with PBS. To keep cells hydrated, a drop of PBS was added to the cells and then exposed to 30 mJ/ cm^2^ dose of UV radiation with an emission peak at 312 nm (in the range of UV-B i.e. 290-320 nm) using UV cross linker (Stratagene) equipped with 5 X 8 W tubes and UV meter (Solar light, Inc.). Media was added after irradiation and cells were incubated for 24 h in previous conditions [37]. WSM (0.05% w/v) was added to cell culture media of 24 h old growing cells before the irradiation and were further incubated at 37°C in a humid atmosphere of 5% CO_2_. Cell morphology was observed by phase contrast microscopy (Olympus). Cell viability was determined using MTT assay.

### Statistical analysis

Each experiment was performed in triplicates unless indicated otherwise. The data are represented as mean ± SD and were compared using Tukey’s test. *p* <0.05 or less was considered to be statistically significant. 

## Results

### WSM is non-cytotoxic to preosteoblasts (MC3T3-E1) and keratinocytes (HaCaT)

**Figure 1:**
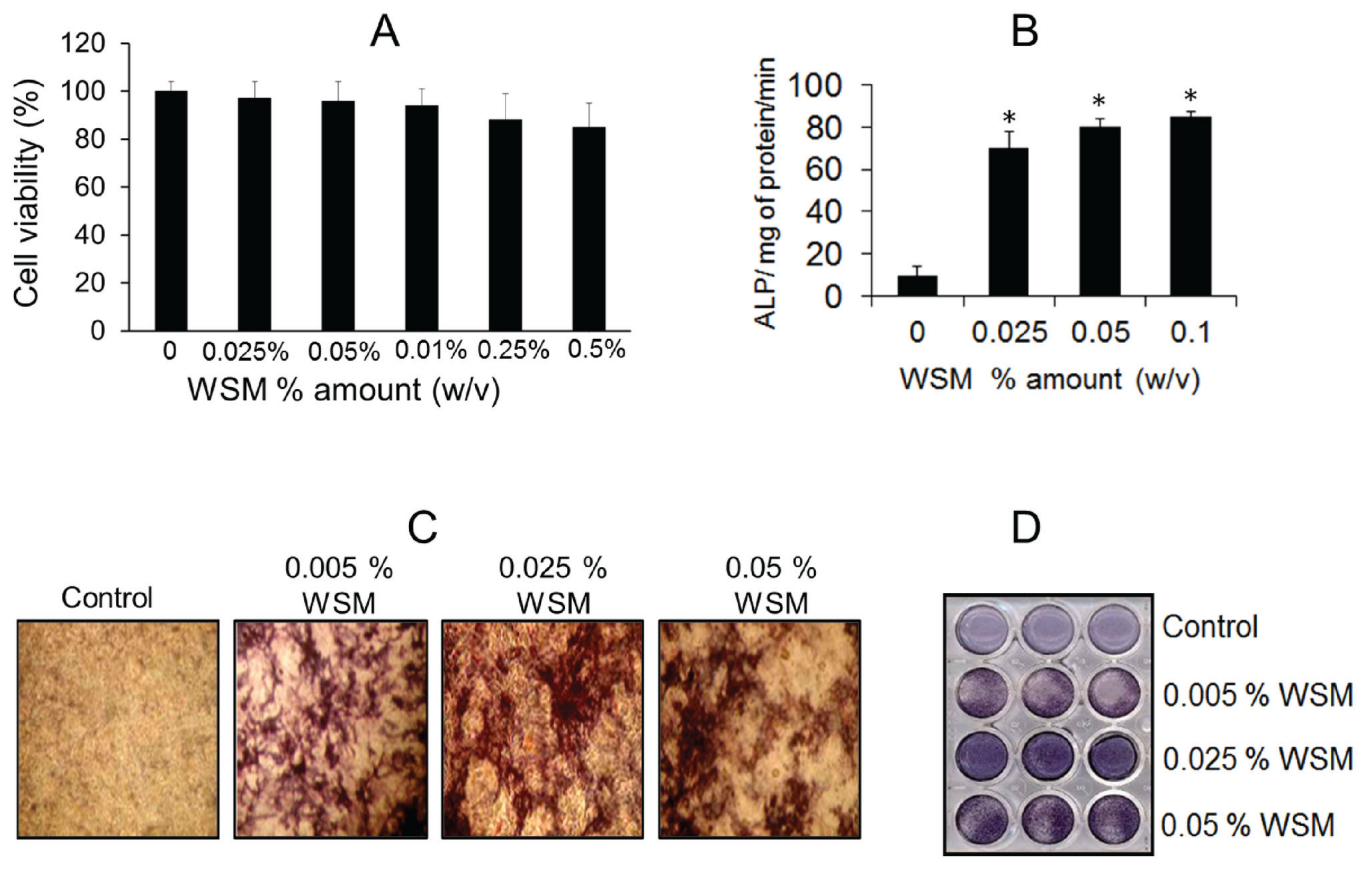
WSM enhances alkaline phosphatase activity (ALP) in MC3T3-E1 cells. **A**: Effect of WSM on cell viability percentage as measured by MTT assay indicating non-toxic nature of WSM for MC3T3-A1 cells. **B**: ALP activity measured by ALP assay kit, shows increases in ALP activity after WSM treatment. Data represent mean -SD. *p ≤ 0.001 (vs. control) where n= 6. **C**: Histochemical staining of MC3T3-A1 cells for ALP activity as observed under phase contrast microscope confirms that WSM increases ALP activity. **D**: One of the plates (out of triplicate) after ALP staining. Results are expressed as mean -SEM (n= 3). *p ≤ 0.05 (vs. control).

To determine the experimental doses of WSM for evaluation of bone differentiation in MC3T3-E1 and UV-protective activity in HaCaT cells, the cytotoxicity of WSM against these cell lines was determined by MTT assay. The cell viability and proliferation rates were not affected by a wide range of concentrations of WSM used and thus indicated its non-cytotoxic nature (Figure **1.A**). Upon 48 h of WSM treatment, the average percentage of cell viability remained similar to the untreated cells (control). Thus 0.025 and 0.05% w/v were chosen for further bioactivity screening studies.

### WSM increases the alkaline phosphatase (ALP) activity

The ALP activity in WSM treated MC3T3-E1 cell line was measured as an early marker of osteoblast differentiation. The ALP activity was increased 7 folds (*p< 0.001*) upon the treatment of 0.025% (w/v) WSM compared to untreated cells (Figure **1.B**). This was further confirmed by activity staining of cellular ALP levels *in vitro*. The WSM treated cells showed significantly (*p< 0.001*) higher staining than the untreated cells (Figure **1.C and D**). 

### WSM increases the transcription of osteoblast differentiation marker genes osteocalcin (OCN) and collagen-1 A2 (COL-1A2)

**Figure 2:**
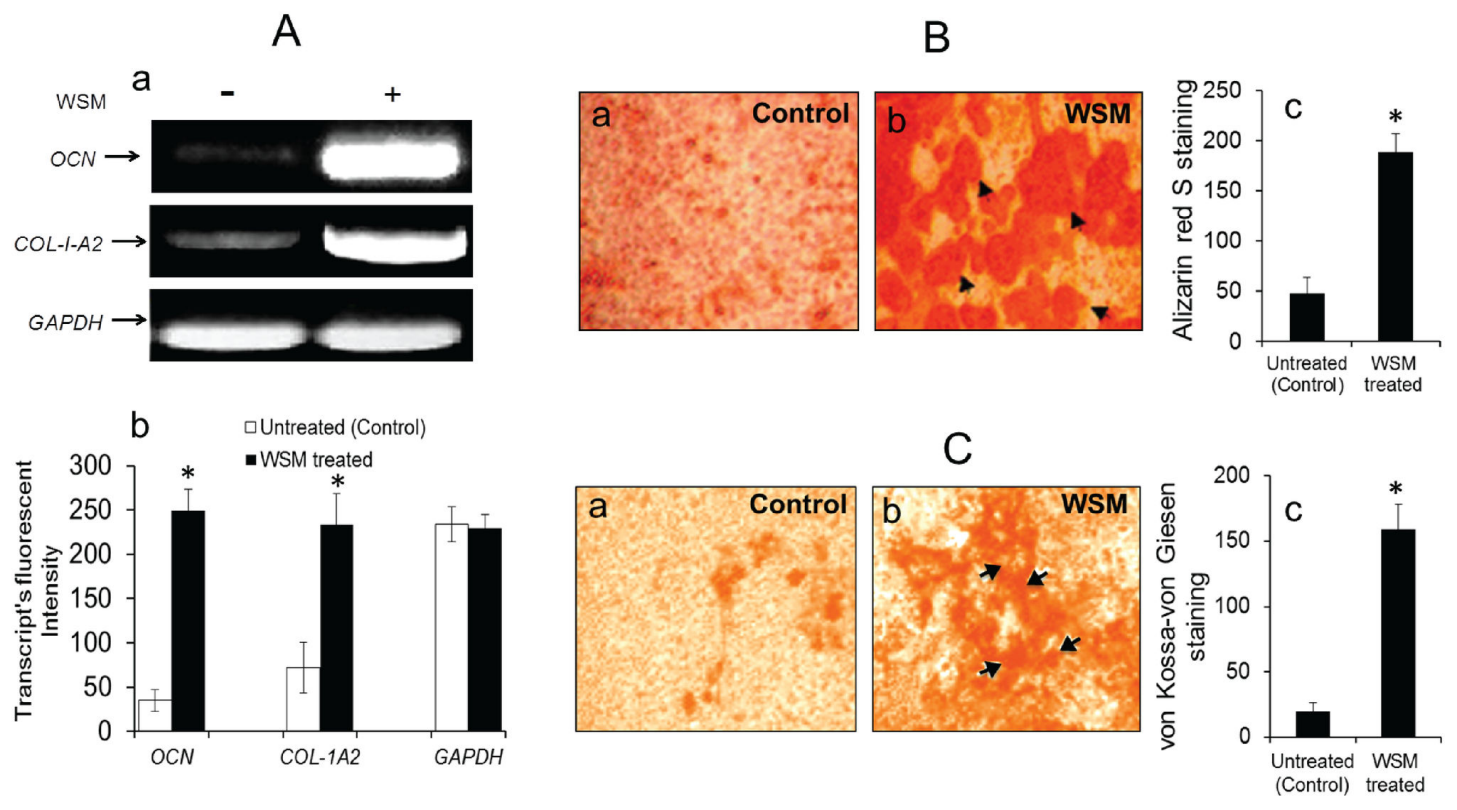
WSM up-regulates osteoblast differentiation markers *OCN* and *COL-1-A2* and accelerates bone nodule formation in MC3T3-A1 cells. **A (a-b)**: Detection of transcriptional levels of osteoblast differentiation marker genes *viz*. osteocalcin (*OCN*) and pro-alpha 2(I) collagen (*COL-1A2*) in RT-PCR of MC3T3-A1 cells, with and without WSM (0.025% w/v) treatment for 24 h. The expression of *GAPDH*, as housekeeping gene was qualitatively (a) detected by the presence of bands in all experiments. Significant increase in transcript (b) of both the marker genes was observed after WSM treatment. **B (a-c)**: Alizarin red S staining of WSM treated (b) and control (a) MC3T3-E1 cells indicating WSM (0.025%, w/v) accelerates calcium deposition and enhances nodule formation. Black arrows indicate the mineral nodules. Quantification (c) of staining intensities were consistent with the visual observation. **C (a-c)**: von Kossa-von Giesen staining of WSM treated (b) and control (a) MC3T3-E1 cells indicating WSM (0.025% w/v) enhances nodule formation. Black arrows indicate the mineral nodules deposition. Quantification (c) of staining intensities were consistent with the visual observation. Results are expressed as mean -SEM (n= 3). *p ≤ 0.05 (vs. control).

The transcriptional expression levels of two osteoblast differentiation markers i.e. osteocalcin (OCN) and collagen-1 A2 (COL-1A2) were measured using a semiquantitative reverse transcriptase-PCR analysis. We observed a significant increase in the transcription of matrix proteins osteocalcin (OCN) and pro-alpha 2(I) collagen (COL-1A2) in MC3T3-E1 cells after 24 h treatment of WSM (Figure **2A**). 

### WSM induced mineralized bone nodule formation

**Figure 3:**
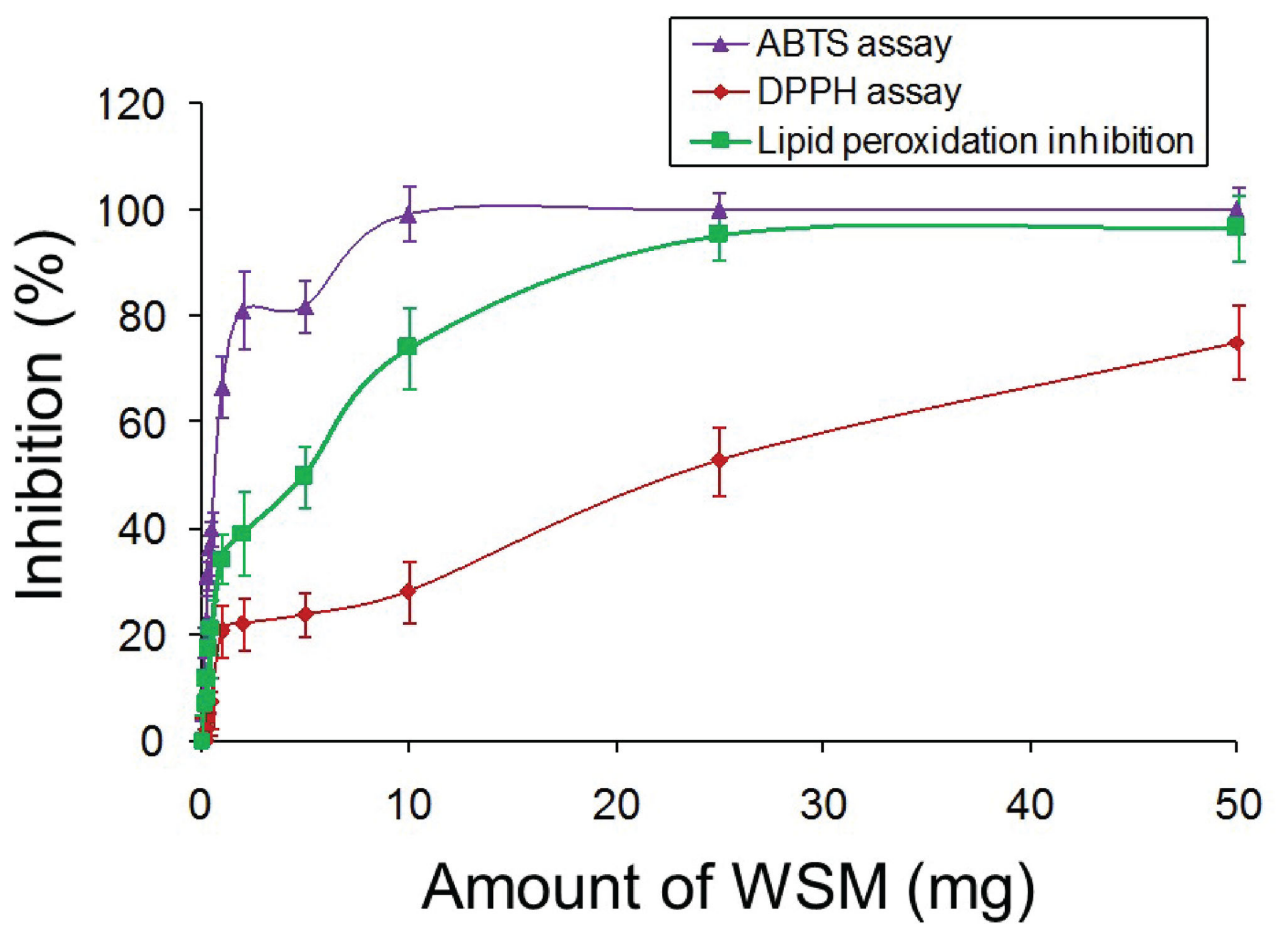
WSM scavenges the ABTS and DPPH free radicals and inhibits lipid peroxidation Percentage inhibition for scavenging capacity of WSM, assayed with three different methods. i.e. ABTS cation decolorization (at 734 nm), DPPH free radical decolorization (at 517 nm) and lipid peroxidation inhibition by measuring the TBARS production (at 532 nm). Results are expressed as mean -SEM (n= 6). *p ≤ 0.05 (vs. control).

Mineralized bone nodule formation indicates the advanced stages of osteoblast differentiation. To test WSM-induced mineralized bone nodule formation in MC3T3-E1 cells, we performed alizarin Red S and von Kossa-von Giesen staining (Figure **2** **. B and C**). The mineralized nodules appeared in cells treated with 0.025% (w/v) of WSM and were stained heavily with alizarin red S (Figure **2.B**). The positive alizarin red S staining indicated the presence of calcified matrix of calcium phosphate, while the untreated cells showed insignificant or no staining (Figure **2.B**). Similarly, von Kossa staining revealed the phosphate deposits while van-Giesen staining showed presence of secreted collagen matrix in cell culture media treated with 0.025% (w/v) of WSM (Figure **3.C**). However, the untreated cells showed insignificant staining or less staining compared to WSM treated cells (Figure **3.C**). 

### WSM scavenges ABTS and DPPH free radicals and inhibited lipid peroxidation

To substantiate the antioxidant capacity of WSM to scavenge the ABTS and DPPH free radicals we followed the decolorization of ABTS^+^ and DPPH. which indicated its capacity to transfer electrons or hydrogen atoms to inactivate these radical cations. For ABTS^+^ and DPPH. decolorization, IC_50_ values of 0.08 and 2.5% w/v (WSM, respectively, were recorded (Figure **3**). Furthermore, the WSM showed significant (*p< 0.05*) free radical scavenging activity in a dose dependent manner.

Similarly, the capacity of WSM to inhibit lipid peroxidation induced by iron and L-ascorbic acid in Swiss albino mice liver homogenate was evaluated by measuring the oxidation product TBARS by means of spectrophotometry. The results showed that WSM effectively inhibited the lipid peroxidation in mice liver homogenate by inhibiting TBARS formation in a dose dependent manner with an IC_50_ value of 0.5% w/v (Figure **3**).

### WSM reduces H_2_O_2_ and UV-B induced damages in human keratinocytes (HaCaT)

**Figure 4:**
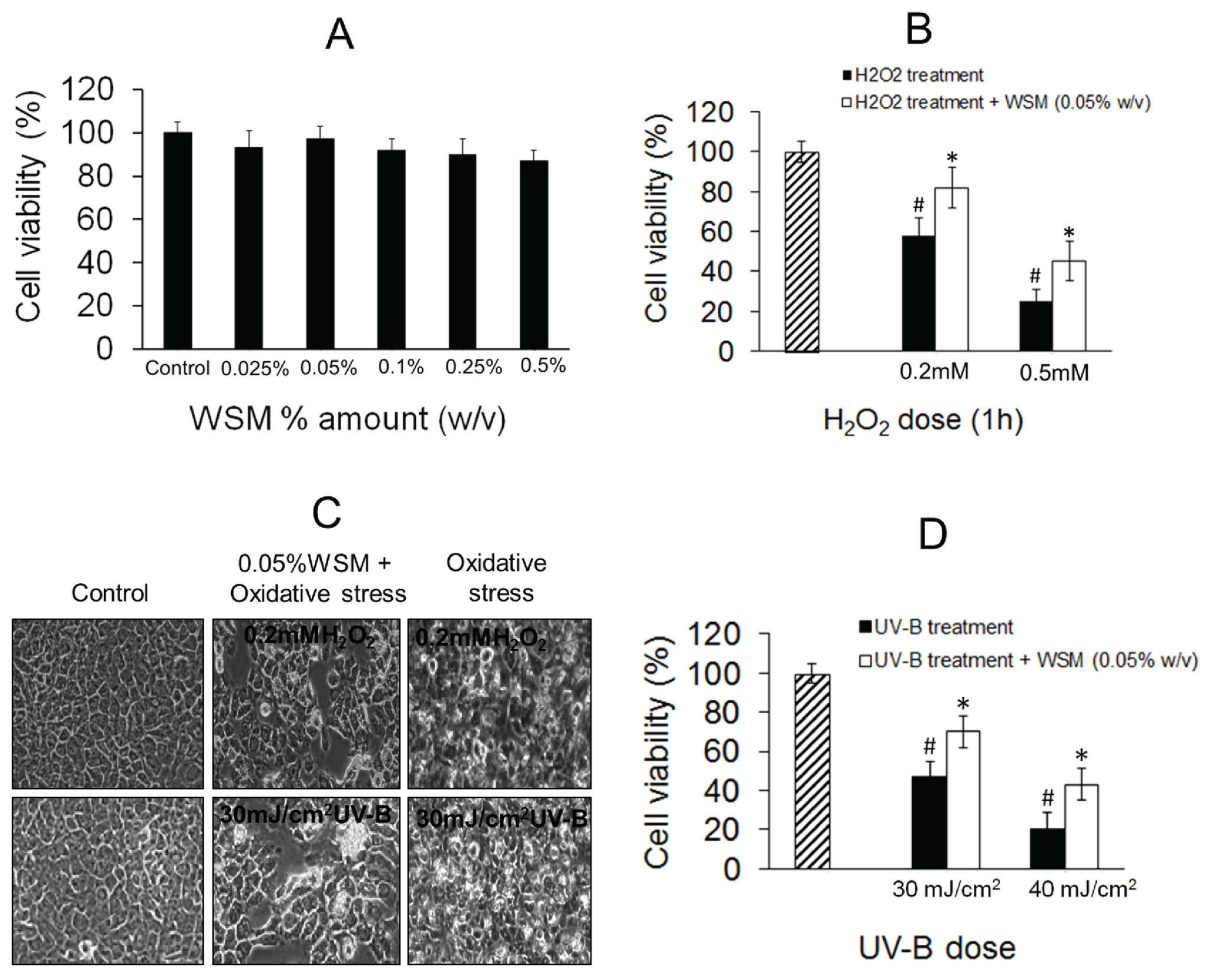
WSM reduces H_2_O_2_ and UV-B induced oxidative damage in HaCaT cells. **A**: Effect of WSM on cell viability percentage as measured by MTT assay indicating non-toxic nature of WSM for HaCaT cells. **B**: Effect of WSM on proliferation of HaCaT cells exposed to H_2_O_2_ (0.25 and 0.5 mM) for 1 h. **C**: Effect of WSM on proliferation of HaCaT cells exposed to UV-B (30 and 40 mJ/ cm^2^). **D**: Effect of WSM on morphogenetic changes in HaCaT cells induced by H_2_O_2_ and UV-B and observed by phase contrast microscopy (200 X). Results are expressed as mean -SEM (n= 6). *p ≤ 0.05 (*vs. control*).

 The antioxidant activity of WSM was investigated in HaCaT cells by inducing oxidative stress using H_2_O_2_ and UV-B irradiation through a cell-based *in vitro* protection assay. Results indicate that WSM significantly inhibited the H_2_O_2_ induced cell death response at a concentration of 0.05% (w/v). H_2_O_2_ treatment of keratinocytes induced oxidative stress, as a result ROS was generated, which induced apoptosis in cells that led to cell damage and eventually, cell death. There was a significant (*p< 0.05*) difference in the cell viability between the control cells, H_2_O_2_ treated cells and WSM + H_2_O_2_ treated cells (Figure **4.A**). Pre-incubation with 0.05% (w/v) WSM, increased the cell viability by 21.2% in 0.25 mM H_2_O_2_ treated keratinocytes (Figure **4.B**). The morphogenetic changes induced by H_2_O_2_ were also shielded by WSM (Figure **4.C**). This result suggests that the effect of H_2_O_2_ induced cell damage and death was reduced after WSM treatment.

Similarly, to determine the effect of WSM on UV-B irradiation induced cell death in keratinocytes, the cells were pre-treated with WSM for 24 h and then irradiated with UV-B (30 mJ/ cm^2^) followed by incubation in culture media for another 24 h. Results suggest that WSM significantly (*p< 0.05*) reduced the UV-B irradiation induced cell death at a dose of 0.05% (w/v) (Figure **4.D**). It was observed that the morphogenetic changes in keratinocyte cells could be shielded by WSM (Figure **4.C**). From these results it was concluded that WSM could inhibit UV-B induced cell death in HaCaT keratinocytes.

## Discussion

The osteoinductive potential of WSM from nacre is well known [3,6,38–40]. However, most of these evidences are limited to studies on fresh water oysters and a very few marine oysters [25,41–48]. Interestingly, in spite of different biomolecular composition of WSM from fresh and marine water oysters, both are able to induce osteoblast differentiation [5,26,49–52]. Several proteins have been identified in WSM with osteoinductive properties indicate their common biochemical nature. This suggests, perhaps the osteoinductive molecules present in nacre act through similar molecular mechanisms. In earlier studies, it was found that oxidative stress inhibits osteoblast differentiation and stimulates bone resorption by activating osteoclasts. It has also been suggested that antioxidant biomolecules could induce osteoblast differentiation [53,54]. Furthermore, the normal differentiation pathway of osteoblastic progenitor cells could be inhibited or altered by lipid or lipoprotein oxidation, which can act as potent inducer of osteoclastic differentiation and thus promotes bone resorption, leading to osteoporosis [55,56]. Therefore an efficient therapeutic approach to reduce the risk of osteoporosis would be to use antioxidant molecules that can suppress lipid oxidation levels [53,57]. Since the WSM proteins are known to be highly charged and tend to deviate from normal protein behavior [24,25,46,58], it is possible that these proteins are able to scavenge or quench the free radicals generated during cellular metabolic responses and therefore are able to fasten the differentiation of osteoblasts. Till date, only handful numbers of studies have investigated the antioxidant potential of the shell powder [17,19,22] and there are no reports available that claim the antioxidant property of WSM. 

Osteogenesis involves the differentiation of mesenchymal cells into pre-osteoblasts, which in turn differentiate into mature osteoblasts, which finally induce the synthesis and deposition of bone matrix proteins leading to bone formation. ALP is widely recognized as an early marker of osteoblast differentiation, which shoots up during differentiation [59]. In our study, WSM treatment enhanced the ALP activity in murine preosteoblast cells (MC3T3-E1) by seven folds, which is a significant increase as compared to untreated cells (control). Moreover, previous studies suggested that collagen bone matrix proteins account for 90% of the total bone matrix proteins while osteocalcin, a non-collagenous protein, is secreted in the later stages of maturation. In our study, we observed the up-regulation of both the genes, i.e. collagen type 1 (COL-1-A2) and osteocalcin (OCN) transcripts. It confirmed that osteoblast differentiation was induced by WSM treatment in MC3T3-E1 cells. At a later stage of differentiation, the osteocalcin protein is known to have high affinity with hydroxyapatite crystals, which regulates nucleation for bone mineralization [60]. In this study, we observed increased osteocalcin transcript levels after WSM treatment, an indication of differentiation of pre-osteoblasts into mature osteoblasts. In addition to this, we also observed increase in von-Kossa staining that stains phosphate deposition and its counter staining with van-Gieson that stains for collagen matrix. These results indicated *in vitro* biomineralization. Moreover, increase in alizarin red S staining which stains for calcium deposits confirmed the presence of hydroxyapatite minerals in the cell culture media after the treatment with WSM. Detection of hydroxyapatite is the indication for initiation of bone formation. Hence, our results suggest that WSM promotes the process of differentiation of pre-osteoblasts into osteoblasts. Osteoblasts secret ALP and collagen type-1 proteins at early stage of differentiation and osteocalcin at later stage, and eventually induce hydroxyapatite nodule formation in the culture medium. In usual mineralizing media, osteoblasts take 21 days to differentiate and form nodules [6], whereas WSM treatment induced bone formation in just 9 days, and therefore this study confirms the osteo-promotive nature of WSM. 

After the confirmation of osteoblast differentiation property of WSM, we investigated the antioxidant potential of WSM to understand the possible means of cellular protection from oxidative stress. Towards this, we measured the antioxidant potential of WSM in cell free media by ABTS and DPPH free radical scavenging assays and lipid peroxidation inhibition methods. All these three different assay conditions resulted in efficient scavenging of different radical species. Although, the values obtained for IC_50_ were not directly comparable, WSM showed similar trends to scavenge all the free radicals in a dose dependent manner. Our results, therefore suggest that WSM is able to scavenge free radicals and hence, poses antioxidant properties. Oxidative stress in biological systems is generally induced by ROS and OH. free radicals, which react with a number of biomolecules such as lipids, proteins and nucleic acids and damage them [61]. Confirmation of antioxidant nature of WSM in cell free medium necessitated the experiments on cell-based protective assays to reduce cellular oxidative stress. The method we used to screen the effect of WSM against H_2_O_2_ and UV-B induced oxidative damages, employed human keratinocyte cells (HaCaT), an well-established system to test antioxidant properties of molecules [36,37]. Indeed, it was observed that WSM did reduce the harmful effects of oxidative stress induced by H_2_O_2_ and UV-B in keratinocytes. These results supported the previous reports about beneficial uses of nacre in skin protection and regenerative therapeutics [20,62]. It has been observed in earlier study that H_2_O_2_ induced oxidative stress reduces the osteoblast differentiation in MC3T3-E1 cells [63]. In a recent study antioxidant peptides from *P. fucata* were found to inhibit the photoaging effect in mice [64]. In addition, enhanced osteogenic differentiation in hydrogel containing nacre powder suggested possible use of nacre in various future tissue-engineering applications [65]. Furthermore, the diversity of shell matrix proteins may be implicated in various biological activities [66]. This study provides strong evidences for the osteogenic and antioxidant properties of WSM, however more detailed study is required to establish the fact that antioxidant molecules of nacre are responsible for osteoblast differentiation. Since WSM is a mixture of molecules further study is required on isolated molecules to evaluate their individual effects. Pending further investigation, the antioxidant nature of WSM molecules is possibly responsible for reducing the osteoclast activity. However, the indications are strong enough in understanding of the holistic effects of WSM on bone physiology and dynamics. Moreover, *in vivo* studies would validate the potential use of WSM from *P. fucata* nacre in development of therapeutic products for bone regeneration and skin protection.
